# Developing and validating measures of self-reported everyday and healthcare discrimination for Aboriginal and Torres Strait Islander adults

**DOI:** 10.1186/s12939-020-01351-9

**Published:** 2021-01-06

**Authors:** Katherine A. Thurber, Jennie Walker, Philip J. Batterham, Gilbert C. Gee, Jan Chapman, Naomi Priest, Rubijayne Cohen, Roxanne Jones, Alice Richardson, Alison L. Calear, David R. Williams, Raymond Lovett

**Affiliations:** 1 National Centre for Epidemiology and Population Health, Research School of Population Health, Australian National University, 54 Mills Road, Acton, ACT 2601 Australia; 2 Centre for Mental Health Research, Research School of Population Health, Australian National University, 63 Eggleston Road, Acton, ACT 2601 Australia; 3 Department of Community Health, Fielding School of Public Health, University of California Los Angeles, 650 Charles E Young Dr S, Los Angeles, CA 90095 USA; 4 Centre for Social Research and Methods, College of Arts and Social Sciences, Australian National University, Ellery Crescent, Acton, ACT, 2601 Australia; 5 Population Health, Murdoch Children’s Research Institute, Royal Children’s Hospital, Flemington Rd, Parkville, Victoria, 3052 Australia; 6 Department of Social and Behavioral Sciences, Harvard T. H. Chan School of Public Health, 677 Huntington Avenue, Kresge Building, Boston, MA 02115 USA; 7 The Australian Institute of Aboriginal and Torres Strait Islander Studies (AIATSIS), 51 Lawson Cres, Acton ACT , 2601 Australia

**Keywords:** Health inequalities, Measurement, Measurement tool development, Psychometrics, Social Epidemitology

## Abstract

**Background:**

It is well established that racism is a fundamental contributor to poor health and inequities. There is consistent evidence of high exposure to discrimination among Aboriginal and Torres Strait Islander (Indigenous Australian) peoples, but impacts have not been fully quantified, in part due to limited measurement tools. We aim to validate instruments developed to measure interpersonal discrimination.

**Methods:**

Instruments were discussed at five focus groups and with experts, and field tested in developing *Mayi Kuwayu: The National Study of Aboriginal and Torres Strait Islander Wellbeing*. Data from 7501 baseline survey participants were analysed. Acceptability was assessed according to extent of missingness, construct validity using exploratory and confirmatory factor analysis, and reliability using Cronbach’s alpha. Associations between each instrument and outcomes conceptually understood to be closely (community-level racism) or less closely (family wellbeing) related were quantified to test convergent and discriminant validity.

**Results:**

An 8-item instrument captures experiences of discrimination in everyday life and a 4-item instrument experiences in healthcare, each followed by a global attribution item. Item missingness was 2.2–3.7%. Half (55.4%) of participants reported experiencing any everyday discrimination, with 65.7% attributing the discrimination to Indigeneity; healthcare discrimination figures were 34.1% and 51.1%. Items were consistent with two distinct instruments, differentiating respondents with varying experiences of discrimination. Scales demonstrated very good reliability and convergent and divergent validity.

**Conclusion:**

These brief instruments demonstrate face validity and robust psychometric properties in measuring Aboriginal and Torres Strait Islander adults’ experiences of interpersonal discrimination in everyday life and in healthcare. They can be used to quantify population-level experiences of discrimination, and associated wellbeing consequences, and monitor change.

**Supplementary Information:**

The online version contains supplementary material available at 10.1186/s12939-020-01351-9.

## Introduction

Racism is a fundamental cause of ill health and health inequities globally [1]. Racism is a system of oppression based on the social ranking of groups of people into categories of race, with those lower in the social strata considered inferior and denied access to rights, resources, and opportunities—processes that have clear health consequences [2]. Discrimination is a manifestation of racism at the intrapersonal, interpersonal, and institutional (systemic) level. Given the difficulty in measuring systemic forms, quantitative research has predominantly focused on experiences of interpersonal discrimination [2–4]. Discrimination can occur on the basis of ethnicity, race, Indigeneity, or other characteristics; it is difficult to identify the basis for discrimination, but regardless of the perceived reason for the discrimination, evidence indicates that there are negative outcomes associated with exposure [3, 5–8].

Aboriginal and Torres Strait Islander (Indigenous Australian) stakeholders have identified as a high priority research on the experiences of discrimination, overall and specifically within healthcare [9, 10]. Regardless of the measure used, there is consistent evidence of high exposure to discrimination in this pouplation [11–17]. This is a consequence of ongoing colonisation, which has contributed to the systematic oppression, disempowerment and exclusion of Aboriginal and Torres Strait Islander peoples.

There is extensive international evidence that experiences of discrimination are associated with poorer wellbeing outcomes, such as psychological distress, depression, anxiety, general health, and physical health—including blood pressure and allostatic load [6, 18]. Discrimination is hypothesised to impact wellbeing through multiple direct and indirect pathways [18]. Racism instigates and perpetuates discrimination and trauma as well as influencing the levels, clustering, and impacts of stressors such as those related to employment, housing, and neighbourhood quality. Discrimination therefore influences access to societal resources and socioeconomic opportunities that promote health. Physiological, behavioural, and psychological responses to discrimination also impact health [19].

Discrimination’s prevalence and strength of association with health varies between and within population groups and by outcome [18]. There is some Aboriginal and Torres Strait Islander-specific evidence on negative outcomes linked to discrimination experiences in everyday life, [11–14, 20, 21] and within healthcare [15, 16]. Given the high exposure prevalence and known association with numerous negative health outcomes, discrimination is likely a substantial contributor to ill health for the population, and to inequities compared to the non-Indigenous population. However, discrimination’s full impacts have not been robustly quantified.

Despite research use of over 100 discrimination instruments [22], a 2010 systematic review identified only 24 instruments with published evidence of psychometric validity [7]. Of the identified instruments, all but one were developed within the United States (US) [7]. These instruments may not capture common forms of discrimination experienced by Aboriginal and Torres Strait Islander peoples, given historical and contextual differences [3, 23, 24]. The review [7] identified one instrument developed for Aboriginal and Torres Strait Islander peoples: *Measuring Indigenous Racism Experiences* (MIRE) [25]. MIRE demonstrated good content and psychometric validity, and reasonable construct and convergent validity and acceptability in a localised sample [25]. Further validation of the instrument in a more heterogeneous sample has not occurred [25]. Other discrimination items have been used in surveys with Aboriginal and Torres Strait Islander peoples; however, to our knowledge, no other discrimination instrument has been validated for use with this population.

The Everyday Discrimination Scale (EDS), a nine-item scale developed to measure exposure to overt discrimination among African-American populations, is among the most commonly used measures internationally [7, 22]. Respondents are asked if they have experienced a set of specific manifestations of interpersonal discrimination, and asked to attribute the discrimination to one or more of their personal characteristics (e.g. race, gender, age). A modified EDS was identified as a reliable and valid measure of discrimination for a diverse sample of US First Nations peoples [24]. This indicates a modified EDS has potential validity for use with other Indigenous populations, noting differences in the discrimination experiences between (and within) Indigenous populations. We are unaware of evidence on the validity of the EDS for any other Indigenous population, or of any healthcare discrimination instruments validated for use with an Indigenous population.

There is a clear need for valid measurement of Aboriginal and Torres Strait Islander peoples’ experiences of discrimination, to inform program and policy development to reduce discrimination and associated harms. The aim of this paper is to conduct a psychometric validation of the instruments developed to capture experiences of overt discrimination in *Mayi Kuwayu: The National Study of Aboriginal and Torres Strait Islander Wellbeing*.

## Methods

### Study population

The Mayi Kuwayu Study is a large-scale, national longitudinal study of adults aged ≥16 years. Rolling baseline data collection commenced in 2018, following multiple years of study development. The study employs multi-mode recruitment, with participants recruited via a postal questionnaire, through word-of-mouth or study advertising, and through on-the-ground community researchers in defined sites. Surveys were self-completed on paper or online, completed with an Aboriginal Mayi Kuwayu Study team member over the phone, or completed with the support of an Aboriginal and/or Torres Strait Islander community researcher. The initial data release (Data Release 1.1) includes 7501 Aboriginal and Torres Strait Islander adults whose survey data were processed by 2 July 2019. Details of the study design are provided elsewhere [26].

### Face validation process

The Mayi Kuwayu Study questionnaire includes a combination of (1) established instruments, where existing instruments had evidence of validity with this population; (2) modified instruments, where instruments existed but had no evidence of, or established limitations to, validity with this population; and (3) new instruments developed through an iterative community consultation process [26].

The drafted instruments for capturing experiences of discrimination and racism were trialed and discussed at five focus groups, spanning major cities to remote areas. As part of these focus groups, participants completed the current draft of the Study questionnaire and provided feedback on the questionnaire items verbally and/or in writing on the questionnaire (Lovett R et al.: Aboriginal and Torres Strait Islander culture and wellbeing indicator development in Australia, in preparation).

In parallel with the focus groups, the questionnaire was further refined through field testing and input from other stakeholders with lived experience and/or content expertise (e.g. field researchers, study Chief Investigators, independent researchers).

### Data

All data analysed in the current study are based on self-reported responses to the final baseline questionnaire, with the exception of remoteness, which was derived based on postcode.

**Fig. 1:**
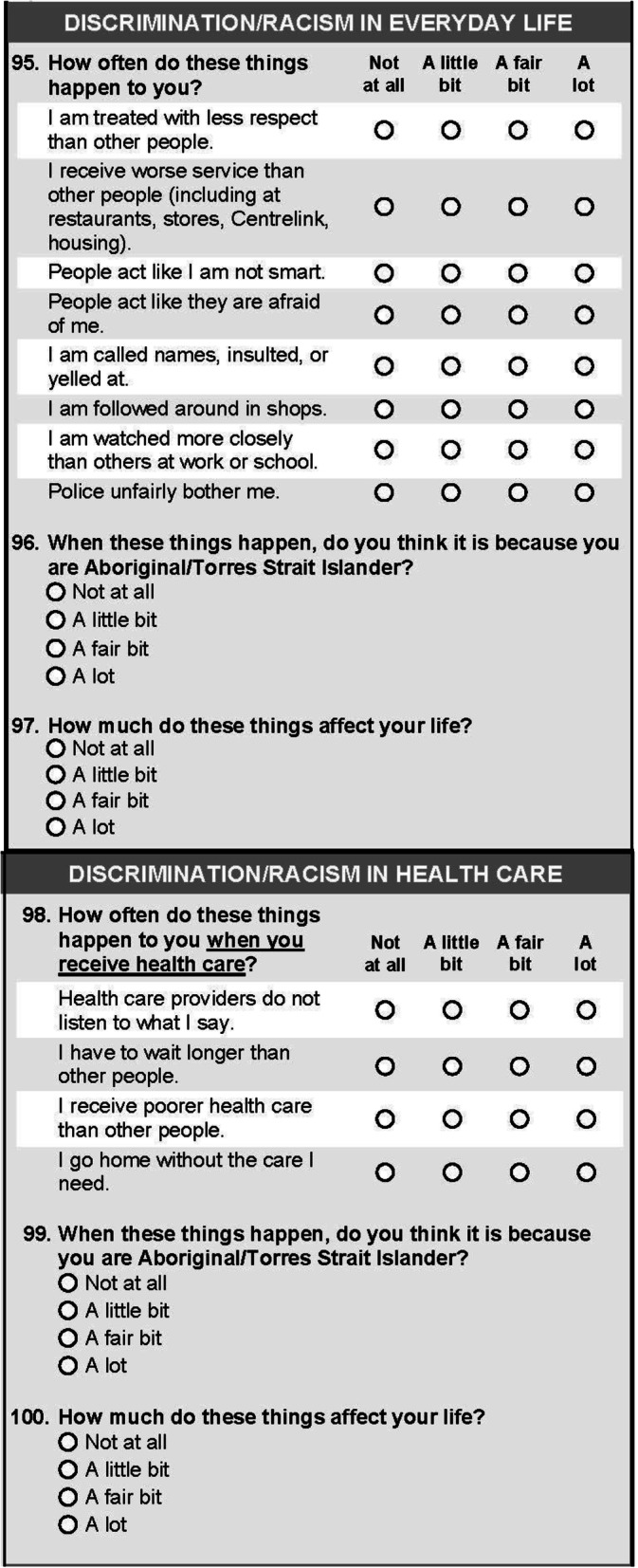
Final version of discrimination measures used in the Mayi Kuwayu Study

For the final discrimination instruments (Fig. 1), response options included “not at all” (coded as 0), “a little bit” (1), “a fair bit” (2), and “a lot” (3). Responses were summed across items to form a total score, which was categorised as no, low, moderate, or high discrimination. Participants with missing data on any item had a missing total score.

After each instrument, participants were asked “When these things happen, do you think it is because you are Aboriginal/Torres Strait Islander?”, with the same response options. For each instrument, participants were categorised as experiencing no discrimination (based on total score, coded as 0), or any discrimination attributed to Indigeneity “not at all” (coded as 1), “a little bit” (2), “a fair bit” (3), or “a lot” (4).

To capture participants’ perceptions of the impacts of discrimination, participants were asked “How much do these things affect your life?”; this variable was treated the same as the above.

Other variables used for validation are described in Additional File 1.

### Statistical analysis

The sample was characterised in relation to age group, gender, and remoteness. For each discrimination instrument, the response distribution across categories was explored for individual items and total scores. Missing data are presented for acceptability assessment. Mean scores (and 95% Confidence Intervals, CIs) are presented overall and by demographic characteristics.

Construct validity was assessed with exploratory factor analysis and Confirmatory Factor Analysis (CFA) for categorical items, each on a random half of the sample. Characteristics of the two sub-samples are summarised in Additional Table 1; chi-squared tests were used to test for differences between groups [27].

**Table 1:** Distribution of responses to discrimination items (*N* = 6775)

	**Mean score (95%CI)**	**% (n)**				
		**Not at all**	**A little bit**	**A fair bit**	**A lot**	**Missing**
**Everyday discrimination**						
I am treated with less respect than other people.	0.53 (0.51,0.55)	59.0 (3994)	29.1 (1971)	6.2 (419)	3.5 (239)	2.2 (152)
I receive worse service than other people (including at restaurants, stores, Centrelink, housing).	0.36 (0.34,0.37)	72.4 (4907)	18.0 (1216)	4.5 (306)	2.6 (179)	2.5 (167)
People act like I am not smart.	0.59 (0.57,0.61)	58.5 (3960)	25.4 (1723)	8.6 (580)	5.1 (345)	2.5 (167)
People act like they are afraid of me.	0.36 (0.34,0.38)	72.1 (4885)	16.8 (1138)	4.8 (324)	2.8 (191)	3.5 (237)
I am called names, insulted, or yelled at.	0.29 (0.27,0.30)	76.5 (5180)	15.9 (1076)	3.1 (208)	1.9 (131)	2.7 (180)
I am followed around in shops.	0.28 (0.26,0.29)	79.3 (5371)	11.6 (785)	3.5 (235)	2.8 (189)	2.9 (195)
I am watched more closely than others at work or school.	0.25 (0.23,0.26)	80.8 (5472)	9.8 (666)	3.1 (207)	2.6 (179)	3.7 (251)
Police unfairly bother me.	0.20 (0.18,0.21)	84.8 (5745)	7.8 (531)	2.2 (148)	2.4 (160)	2.8 (191)
*Total everyday discrimination score*	*2.76 (2.66,2.86)*	*41.8 (2832)*	*43.5 (2946)*	*6.6 (444)*	*1.9 (126)*	*6.3 (427)*
	**% (n)**					
	**No discrimination**	**Not at all**	**A little bit**	**A fair bit**	**A lot**	**Missing**
When these things happen, do you think it is because you are Aboriginal/Torres Strait Islander?	41.8 (2832)	19.1 (1291)	20.9 (1417)	7.6 (512)	8.0 (543)	2.7 (180)
How much do these things affect your life?	41.8 (2832)	20.2 (1370)	24.0 (1626)	6.7 (456)	4.7 (319)	2.5 (172)
**Healthcare discrimination**						
Health care providers do not listen to what I say.	0.39 (0.38,0.41)	69.4 (4699)	20.0 (1352)	4.6 (311)	3.0 (203)	3.1 (210)
I have to wait longer than other people.	0.23 (0.22,0.25)	81.1 (5496)	10.5 (714)	3.0 (201)	2.0 (133)	3.4 (231)
I receive poorer health care than other people.	0.18 (0.17,0.19)	84.3 (5708)	8.4 (570)	2.2 (148)	1.6 (106)	3.6 (243)
I go home without the care I need.	0.26 (0.25,0.28)	79.1 (5362)	11.8 (800)	2.9 (194)	2.7 (180)	3.5 (239)
*Total healthcare discrimination score*	*1.06 (1.00,1.11)*	*63.1 (4277)*	*26.7 (1811)*	*4.3 (291)*	*1.7 (116)*	*4.1 (280)*
	**% (n)**					
	**No discrimination**	**Not at all**	**A little bit**	**A fair bit**	**A lot**	**Missing**
When these things happen, do you think it is because you are Aboriginal/Torres Strait Islander?	63.1 (4277)	16.5 (1115)	11.3 (768)	2.9 (196)	3.0 (200)	3.2 (219)
How much do these things affect your life?	63.1 (4277)	11.5 (777)	14.0 (947)	4.2 (285)	3.7 (253)	3.5 (236)

*For individual discrimination items, response options included “not at all” (coded as 0), “a little bit”* (1)*, “a fair bit”* (2)*, and “a lot”* (3)*. Responses were summed across items (range:0–24 for everyday and 0–12 for healthcare discrimination) to form a total score. Participants with missing data on any individual item had a missing total score. A categorical total score variable was derived for each measure, designed to align with the scoring for individual items: no discrimination (score 0; presented as “not at all” in this table), low discrimination (1–8 for everyday and 1–4 for healthcare discrimination; presented as “a little bit” in this table), moderate discrimination (9–16 and 5–8, respectively; presented as “a fair bit” in this table), and high discrimination (17–24 and 9–12, respectively; presented as “a lot” in this table)*

Principal Axis Factor (PAF) was applied to the “developmental” sub-sample to test the factor structure and identify poorly fitting items for potential removal. Based on the exploratory findings, CFA was conducted in the “validation” sub-sample to confirm unidimensionality and test model fit. Indicators of model fit and appropriateness are described in Additional File 1 [28–33].

The relationship between the latent variable and item responses was assessed using a generalised partial credit model for ordinal items. Item slope coefficients 0.5–2.0 are expected [34], with higher values indicating greater discernment between participants on the latent variable; coefficients >4.0 indicate too much covariation with other items [35]. Difficulty thresholds identify the point on the latent variable at which a response category is more likely to be endorsed than the previous category. Non-overlapping 95% CIs between successive difficulty thresholds indicate a linear progression of response categories.

In each sub-sample, reliability of each instrument was assessed using Cronbach’s alpha (α≥0.7 and <0.90) [36].

We tested convergent and discriminant validity by quantifying the association between each discrimination instrument (categorical) and an outcome conceptually understood to be closely related (problems with racism in the community), and an outcome conceptually understood to be less closely related (family wellbeing). We expected to see a stronger association between the discrimination scales and community-level racism (positive), compared to family wellbeing (negative). We calculated prevalence ratios (PRs) and 95%CIs using log-binomial models. We calculated correlation coefficients to assess the continuous relationships, excluding participants missing discrimination scores. Correlations of magnitude 0.10–0.29 were considered “weak”, 0.30–0.49 “moderate”, and 0.50–1.00 “strong” [37].

An alpha level of 0.05 was the threshold for statistical significance.

## Results

### Face validity

The initial racism instrument tested through the Mayi Kuwayu Study was adapted from the first question of the MIRE (Table S1). MIRE uses a one-stage approach to ask about experiences of unfair treatment due to being Aboriginal and/or Torres Strait Islander [25]. This instrument was tested in two remote settings and discussed in-depth during one focus group. Researchers conducting the focus group identified that many participants described experiences where they were treated unfairly, but because these experiences were so common, it was perceived as "normal" treatment and not identified as discrimination. Alternative instruments were tested in the next survey version, aiming to elicit responses about specific circumstances.

A two-stage approach was employed next (Table S2), first asking about any experiences of discrimination, and then asking if each experience was attributed to Indigenous status. It included items capturing experiences of everyday (13 items) and healthcare (7 items) discrimination. Questions were adapted from previous instruments, including the EDS, or developed based on literature or personal experiences. The response options were changed to match those received positively throughout Mayi Kuwayu Study engagement/feedback processes (“not at all”, “a little bit”, “a fair bit”, “a lot”). Respondents were asked to tick “Yes” for each item if they considered the treatment was due to their Indigeneity. After each set of items, participants were asked two questions about the discrimination’s perceived impact. This version was tested in two major cities. There was heterogeneity in responses, but very few respondents assigned attribution. Field researchers expressed concern that respondents were inadvertently skipping the attribution question due to the multi-column formatting.

The items were restructured into a single-column format in the subsequent survey version. Due to space constraints, a single global attribution question was used, covering all items in the instrument. The question “How stressful is it when these things happen?” was removed, and the wording for the final question was changed to “How much do these things affect your life?”. This version was trialed in one inner regional area. The instruments were subsequently reduced through consultation; the final survey included an 8-item everyday and 4-item healthcare discrimination instrument (Fig. 1).

### Psychometric validation

The sample included 6775 adults after excluding those missing age, gender, or remoteness (9.7% excluded). 61.3% of the sample was female, 45.8% from major cities, and 63.8% aged ≥46 years; the distribution of the sample differs from that of the total Aboriginal and Torres Strait Islander adult population (Table S3).

Across items, 2.2–3.7% of respondents did not provide a response, indicating the items were broadly acceptable (Table 1). The majority of the sample (58.5–84.8%) reported no experience of each discrimination item.

For the everyday discrimination instrument, the most highly endorsed items were "People act like I am not smart" (mean 0.59;95%CI:0.57,0.61) and "I am treated with less respect than other people" (mean 0.53;0.51,0.55). The mean total score was 2.76 (2.66,2.86), with 41.8% experiencing no, 42.5% low, 6.6% moderate, and 1.9% high discrimination. For the healthcare discrimination instrument, the highest endorsed item was “Health care providers do not listen to what I say” (mean: 0.39;0.38,0.41). The mean total score was 1.06 (1.00,1.11), with 63.1% experiencing no discrimination, 26.7% low, 4.3% moderate, and 1.7% high discrimination. For both instruments, the majority of those experiencing discrimination made some attribution to Indigeneity, and around two-thirds reported that their experiences affected their life “a little” to “a lot”.

The mean total score for everyday and healthcare discrimination varied significantly by age group (lowest among those aged ≥66 years) and remoteness (highest in remote and very remote areas); scores did not vary significantly by gender (Table S4).

PAF results were consistent with two distinct factors, aligned with the intended instruments, with all factor loadings >0.3 and negligible cross-loadings (Table 2). Within this sub-sample, Cronbach’s α was 0.894 for everyday and 0.853 for healthcare discrimination, indicating very good internal consistency.

**Table 2:** PAF analysis for discrimination items combined (two factors), and individually, within the “developmental” sub-sample

Measure	Item	Factor	
		1	2
Everyday	I am treated with less respect than other people.	0.745	
Everyday	I receive worse service than other people …	0.750	
Everyday	People act like I am not smart.	0.736	
Everyday	People act like they are afraid of me.	0.787	
Everyday	I am called names, insulted, or yelled at.	0.666	
Everyday	I am followed around in shops.	0.759	
Everyday	I am watched more closely than others at work or school.	0.694	
Everyday	Police unfairly bother me.	0.609	
Health system	Healthcare providers do not listen to what I say.		0.711
Health system	I have to wait longer than other people.		0.717
Health system	I receive poorer health care than other people.		0.873
Health system	I go home without the care I need.		0.792

The CFA results confirmed the unidimensional nature of each instrument. For the everyday and healthcare discrimination instruments, the standardised coefficients loaded significantly onto a single dimension and ranged from 0.60–0.79 and 0.71–0.86, and fit was deemed to be acceptable (SRMR = 0.04 and 0.03, CFI = 0.94 and 0.98, TLI = 0.92 and 0.94). The RMSEA indicated model misfit (0.10 and 0.15), but the SRMR was prioritised given item skewness.

For everyday discrimination items, no coefficients were indicative of excessive item covariation; the lowest coefficient was for “Police unfairly bother me” (1.59) (Table 3). The response categories for the item “People act like I am not smart” showed an upward progression with non-overlapping CIs between category thresholds. Category threshold parameters were not in sequential order for the police item, indicating a potential issue with participants’ response grading. The remaining six items had overlapping CIs between category thresholds “a little bit and a fair bit” and “a fair bit and a lot” suggesting these response categories may have been difficult to distinguish.

**Table 3:** Item discrimination and difficulty parameters for everyday and healthcare discrimination items, within the “validation” sub-sample

	Item discrimination parameter (standard error)	Category threshold parameter (95% CI)		
		“Not at all” and “A little bit”	“A little bit” and “A fair bit”	“A fair bit” and “A lot”
**Everyday discrimination items**				
1. I am treated with less respect than other people.	2.64 (0.12)	0.41 (0.36, 0.47)	1.53 (1.44, 1.62)	1.69 (1.58, 1.80)
2. I receive worse service than other people (including at restaurants, stores, Centrelink, housing).	3.06 (0.16)	0.82 (0.76, 0.87)	1.61 (1.52, 1.70)	1.78 (1.66, 1.89)
3. People act like I am not smart.	2.62 (0.13)	0.43 (0.37, 0.48)	1.29 (1.21, 1.37)	1.55 (1.45, 1.65)
4. People act like they are afraid of me.	1.93 (0.10)	1.05 (0.96, 1.13)	1.70 (1.58, 1.81)	1.80 (1.64, 1.96)
5. I am called names, insulted, or yelled at.	1.92 (0.10)	1.19 (1.10, 1.29)	1.97 (1.83, 2.11)	1.84 (1.65, 2.03)
6. I am followed around the shops.	2.27 (0.13)	1.25 (1.16, 1.34)	1.75 (1.62, 1.87)	1.67 (1.52, 1.82)
7. I am watched more closely than others at work or school.	2.61 (0.16)	1.33 (1.24, 1.43)	1.67 (1.56, 1.79)	1.69 (1.55, 1.83)
8. Police unfairly bother me.	1.59 (0.10)	1.96 (1.78, 2.14)	1.92 (1.73, 2.11)	1.52 (1.29, 1.75)
**Healthcare discrimination items**				
1. Health care providers do not listen to what I say.	2.56 (0.15)	0.72 (0.66, 0.78)	1.59 (1.49, 1.69)	1.69 (1.57, 1.82)
2. I have to wait longer than other people.	3.14 (0.21)	1.14 (1.07, 1.22)	1.69 (1.58, 1.79)	1.85 (1.71, 1.99)
3. I receive poorer health care than other people.	5.21 (0.45)	1.18 (1.12, 1.24)	1.66 (1.57, 1.75)	1.91 (1.79, 2.03)
4. I go home without the care I needed.	3.36 (0.23)	1.04 (0.97, 1.11)	1.60 (1.51, 1.70)	1.66 (1.55, 1.78)

For the healthcare items, the slope coefficient for “I receive poorer health care than other people” (5.21) indicated excessive covariance (Table 3). Response categories for this item showed a linear upward progression with non-overlapping CIs; for the other three items, CIs overlapped across categories “a little bit and a fair bit” and “a fair bit and a lot”, as above.

In the “validation” sub-sample, Cronbach’s α was 0.891 for everyday discrimination and 0.857 for healthcare discrimination, indicating very good internal consistency.

Both instruments demonstrated convergent validity with community-level racism (Table 4). The prevalence of any community-level racism was significantly higher among those experiencing low, moderate, or high, compared to no, discrimination in everyday life (PR = 3.30;2.95,3.69 for high) or healthcare (PR = 1.88;1.62,2.16 for high). Correlation with community-level racism was moderate (0.41) for everyday, and weak (0.26) for healthcare discrimination total score. Associations were of reduced magnitude, though still significant, for family wellbeing, supporting divergent validity.

**Table 4:** Association between discrimination scales and psychological distress (convergent validity) and family wellbeing (divergent validity)

	Any problems with racism in the community		High family wellbeing	
	% (n/N)	PR (95%CI)	% (n/N)	PR (95%CI)
**Everyday discrimination score**				
Not at all	24.6 (614/2500)	1 (Ref)	55.1 (1091/1982)	1 (Ref)
A little bit	57.5 (1546/2687)	2.34 (2.17,2.53)	40.8 (957/2348)	0.74 (0.69,0.78)
A fair bi	78.3 (325/415)	3.19 (2.93,3.47)	32.4 (119/367)	0.59 (0.50, 0.68)
A lot	81.0 (94/116)	3.30 (2.95,3.69)	41.5 (44/106)	0.75 (0.59, 0.94)
Missing	51.3 (143/279)	2.09 (1.83,2.38)	42.1 (107/254)	0.77 (0.65, 0.88)
**Healthcare discrimination score**				
Not at all	36.1 (1379/3825)	1 (Ref)	50.5 (1595/3157)	1 (Ref)
A little bit	61.0 (1018/1668)	1.69 (1.60,1.79)	38.4 (555/1446)	0.76 (0.70, 0.81)
A fair bit	72.7 (194/267)	2.02 (1.85,2.19)	33.1 (77/233)	0.65 (0.54, 0.78)
A lot	67.7 (67/99)	1.88 (1.63,2.16)	33.7 (31/92)	0.67 (0.49, 0.89)
Missing	46.4 (64/138)	1.29 (1.07,1.55)	46.5 (60/129)	0.92 (0.76,1.11)

*Restricted to participants with data on the outcome of interest. All models were unadjusted, and did not take into account potential geographic clustering in the sample, as the aim of this analysis was to test if the hypothesised association exists, rather than to quantify the magnitude of the association in the cohort*

## Discussion

These instruments, capturing discrimination experiences in everyday life and in healthcare, were assessed on their face value to capture experiences of Aboriginal and Torres Strait Islander peoples, using robust and iterative consultative processes. The instruments have robust psychometric properties: acceptability, internal consistency, and construct validity. They are relatively brief, and can be used independently or in combination. The instruments capture broad experiences of discrimination, and additional items enable researchers to identify attribution to Indigeneity. They complement the current single other validated discrimination instrument for Aboriginal and Torres Strait Islander peoples, the MIRE [25].

The final instruments performed relatively well in terms of unidimensional fit. The healthcare discrimination item “I receive poorer health care than other people” may have been too broad and somewhat redundant. The additional response burden of answering this item was considered minimal and the item was therefore retained.

A strength of these discrimination instruments is the multiple approaches employed to generate, modify, and validate the instruments—with active involvement of Aboriginal and Torres Strait Islander peoples at all stages. Items were informed by the literature and adapted from previous instruments. New items specific and acceptable to the Aboriginal and Torres Strait Islander population were generated through consulting community members and researchers with content expertise. Face and content validation approaches were iterative and integrated input from the target population and “expert judges” [30], and included focus groups, pilot testing with diverse members of the target population, and review by multiple stakeholder groups. These qualitative processes identified issues with item interpretation and format, and culminated in instruments considered acceptable to the stakeholders involved.

The two-stage approach of measuring all forms of discrimination and identifying experiences attributed to Indigeneity offers data users flexibility in data use. Given difficulties accurately attributing discrimination experiences, the true prevalence of interpersonal discrimination due to Indigeneity likely lies between the prevalence of any discrimination and that attributed to Indigeneity [5]. Some experiences of discrimination due to Indigeneity would likely be missed using a one-stage approach.

The final item about discrimination’s impacts enables examination of participants’ self-identified impacts of their discrimination experiences.

### Strengths and limitations

These scales are promising as measures of self-reported experiences of discrimination in everyday life and in healthcare among the Aboriginal and Torres Strait Islander adults (≥16 years). Generalisability of these findings beyond the cohort is unknown; however, the Mayi Kuwayu Study cohort is heterogeneous. The large sample size, and more than satisfactory subject-to-item ratio (>500), [7, 27, 30] would enable examination of the measure by different groups (e.g. by age group, gender, geography, or other characteristic) in future research, including further exploration of the value of including the item on “poorer treatment” in healthcare.

While the scales used in the Mayi Kuwayu Study were adapted from the EDS and other measures, modifications were substantial in order to be relevant and appropriate for the population of interest; this reduces comparability across populations.

In the absence of a gold standard measure of experiences of discrimination, precluding examination of criterion validity [30], scale scores were correlated against measures theorised to be associated with the construct [27].

The instruments used in the Mayi Kuwayu Study do not refer to experiences of discrimination over a specified time period; rather, they capture lifetime exposure. The instruments do not use objective measures of frequency (i.e. weekly or daily), as previous research has demonstrated that subjective assessment of frequency (as occurs here) can be easier than quantification [38].

Future work could explore the implications of using the instruments in continuous versus categorical form, and identify relevant cut-offs specific to this population. The four-tiered response category may have been unnecessary in this sample; future research could explore if fewer response categories would suffice.

While the terms “racism” and “discrimination” were not used within the instruments, these words were made salient through their use in the survey section header [7]. This could have contributed to increased reporting of experiences of discrimination and/or increased attribution to Indigeneity, compared to a neutral header [7].

These instruments are based on self-reported experiences of discrimination, and self-identification of attribution, which may induce biases such as minimisation bias and vigilance bias [6]. However, experiences of discrimination are subjective (this does not mean they are not real), and therefore reliance on self-report is appropriate [3, 6–8, 35].

The instruments developed are not exhaustive. As with any measure, they will not capture all experiences of discrimination in everyday life, or in healthcare [7]. Further, these scales do not capture experiences of systemic discrimination, which are difficult to capture by asking about specific individual experiences, [3, 7, 8] but are acknowledged to be common [4, 39]. Methodological advances are required to enable survey research to capture systemic discrimination.

## Conclusions

Racism is a fundamental contributor to ill health and to health inequities for Aboriginal and Torres Strait Islander peoples. High quality measurement of experiences of discrimination is therefore essential to underpin action to improve health and reduce inequities.

These instruments capture Aboriginal and Torres Strait Islander peoples’ experiences of interpersonal discrimination. They can be used to enable valid measurement of discrimination’s prevalence, in order to identify priority targets for action, quantify discrimination’s contribution to health and health inequities, monitor trends, and evaluate interventions.

The instruments may be meaningful for use with other Indigenous populations with similar discrimination experiences; however, cross-cultural validity would need to be explored, and local adaptation may be required.

## Supplementary Information

**Additional file 1.**

## Data Availability

The datasets analysed during the current study are available on application to the Mayi Kuwayu Study Data Governance Committee. This governance body oversees and approves applications for data use, in order to maintain the confidentiality of participants, and ensure that all studies using the Mayi Kuwayu data are protective of Aboriginal and Torres Strait Islander data and cultures. The data application process is detailed here: mkstudy.com.au/overview/.

## Abbreviations

US: United States
MIRE: Measuring Indigenous Racism Experiences
EDS: The Everyday Discrimination Scale
PAF: Principal Axis Factor
CFA: Confirmatory Factor Analysis
PRs: Prevalence Ratios
HRECs: Human Research Ethics Committees
SE: Standard Error
CIs: Confidence Interval
RMSEA: Root mean square error of approximation
SRMR: Root mean squared residual
CFI: Comparative fit index
TLI: Tucker-Lewis Index
