# Supplementary material for: Developing and validating measures of self-reported everyday and healthcare discrimination for Aboriginal and Torres Strait Islander adults
Source: Int J Equity Health. 2021 Jan 6;20:14. doi: 10.1186/s12939-020-01351-9 (PMC7788827; doi:10.1186/s12939-020-01351-9)
Supplement: Supplementary file 1 — Additional file 1. [file 12939_2020_1351_MOESM1_ESM.docx]

# Supplementary (additional) information

**Additional File 1: details on variable definitions**

Age, calculated from date of birth and date of survey completion, was categorised into 10-year groups starting from age 16, up to 65 years (16-25, 26-35, 36-45, 46-55, 56-65, ≥66 years). Indigenous identification was reported as Aboriginal, Torres Strait Islander, or Aboriginal and Torres Strait Islander. Gender was based on identification as male, female, or other. Due to small numbers of persons identifying as other genders, this category is included in totals but is omitted from gender-stratified and adjusted analysis.

Problems with racism in the community is measured according to participants’ responses to the question, “Are any of these a problem where you live? … Racism?” The response options were “not at all” (coded as 1), “a little bit” (2), “a fair bit” (3), and “a lot” (4). A binary variable was created, indicating that racism is not a problem in the community (1) versus “a little bit” to “a lot” of a problem (2-4).

Family wellbeing was measured using a scale created through the Mayi Kuwayu Study development process. The validity of this scale has not yet been validated. Participants were asked to rate the extent to which they agree with a set of 9 statements starting with the stem, “In my family…”, such as “we get on together and cope in the hard times” and “we celebrate special days/events”. Response options were: “not at all” (coded as 1), “a little bit” (2), “a fair bit” (3), “a lot” (4), or “unsure” (recoded as missing). When there are responses to all of the items in the scale, responses are summed to generate a total family wellbeing score ranging from 9 to 36. A categorical variable was created based on the sample distribution, with a score 9-23 reflecting low, 24-29 moderate, 30-34 high, and 34-36 very high family wellbeing. A binary variable was created, of low or moderate (9-29) versus high (30-36) family wellbeing.

**Additional File 2: details on assessment of model fit and appropriateness**

Principal Axis Factor (PAF) was applied to the “developmental” sub-sample to test the factor structure and identify poorly fitting items for potential removal. Appropriateness of PAF was evaluated using Pearson correlation coefficient (items |r|<0.4 or >0.8 potentially unsuitable),^1^ Bartlett’s test of sphericity (p-value<0.05), and Kaiser-Meyer-Olkin (KMO>0.6 suitable).^2^ PAF was conducted on the two instruments combined, setting two factors and using oblique rotation, to test if the instruments were measuring independent constructs. The number of components for extraction was determined through visual examination of the scree plot. Cross-loadings >0.30 and factor loadings <0.30 were considered problematic.^3^

Based on the exploratory findings, Confirmatory Factor Analysis (CFA) was conducted in the “validation” sub-sample to confirm unidimensionality and test model fit. Item standardised coefficients were assessed to identify poor fitting items, according to root mean square error of approximation (RMSEA≤0.06 for good model fit), root mean squared residual (SRMR ≤0.08), and comparative fit index (CFI) and Tucker-Lewis Index (CIF and TLI ≥0.95 for good model fit, 0.90-0.95 acceptable fit).^4,5^ If the fit indices disagreed due to data skewness, the SRMR was prioritised over the RMSEA.^6^

**SI Table 1. Characteristics of participants in the exploratory and confirmatory factor analysis sub-samples**

|  | Sub-sample | | |  | Total  N=6775 |
| --- | --- | --- | --- | --- | --- |
|  | EFA  N=3387 |  | CFA  N=3388 |  |  |
|  | % (n) |  | % (n) |  | % (n) |
| Indigenous identification |  |  |  |  |  |
| Aboriginal | 91.5% (3098) |  | 92.2% (3123) |  | 91.8% (6221) |
| Torres Strait Islander | 3.2% (109) |  | 3.0% (101) |  | 3.1% (210) |
| Both Aboriginal and Torres Strait Islander | 4.3% (145) |  | 3.7% (126) |  | 4.0% (271) |
| Missing | 1.0% (35) |  | 1.1% (38) |  | 1.1% (73) |
| Age group |  |  |  |  |  |
| 16-25 years | 10.9% (368) |  | 9.7% (327) |  | 10.3% (695) |
| 26-35 years | 12.0% (407) |  | 11.9% (402) |  | 11.9% (809) |
| 36-45 years | 14.0% (475) |  | 14.0% (475) |  | 14.0% (950) |
| 46-55 years | 20.5% (696) |  | 20.2% (685) |  | 20.4% (1381) |
| 56-65 years | 25.5% (863) |  | 25.3% (856) |  | 25.4% (1719) |
| 66+ years | 17.1% (578) |  | 19.0% (643) |  | 18.0% (1221) |
| Level of remoteness |  |  |  |  |  |
| Major Cities | 45.7% (1547) |  | 45.8% (1553) |  | 45.8% (3100) |
| Inner Regional areas | 29.0% (983) |  | 28.6% (970) |  | 28.8% (1953) |
| Outer regional areas | 18.2% (617) |  | 17.9% (606) |  | 18.1% (1223) |
| Remote and very remote areas | 7.1% (240) |  | 7.6% (259) |  | 7.4% (499) |
| Everyday discrimination |  |  |  |  |  |
| No discrimination (score=0/24) | 41.1% (1392) |  | 42.5% (1440) |  | 41.8% (2832) |
| Low discrimination (score 1-8/24) | 44.0% (1490) |  | 43.0% (1456) |  | 43.5% (2946) |
| Moderate discrimination (score 9-16/24) | 6.9% (234) |  | 6.2% (210) |  | 6.6% (444) |
| High discrimination (score 17-24/24) | 2.0% (67) |  | 1.7% (59) |  | 1.9% (126) |
| Missing | 6.0% (204) |  | 6.6% (223) |  | 6.3% (427) |
| Healthcare discrimination |  |  |  |  |  |
| No discrimination (score=0/12) | 63.4% (2146) |  | 62.9% (2131) |  | 63.1% (4277) |
| Low discrimination (score 1-4/12) | 26.8% (907) |  | 26.7% (904) |  | 26.7% (1811) |
| Moderate discrimination (score 5-8/12) | 4.3% (144) |  | 4.3% (147) |  | 4.3% (291) |
| High discrimination (score 9-12/12) | 1.6% (55) |  | 1.8% (61) |  | 1.7% (116) |
| Missing | 4.0% (135) |  | 4.3% (145) |  | 4.1% (280) |

No significant differences were observed between groups, with the p-value for all chi-squared tests ≥0.05.
Due to small numbers of persons identifying as other gender, this group is included in the sub-samples but is omitted from the table.

Table S1. First version of discrimination instruments tested in the Mayi Kuwayu Study

| **Prompt: We want to find out about times when you were treated unfairly *because you are an Aboriginal or Torres Strait Islander person*. This includes being treated rudely, with disrespect, or as if you were inferior; being ignored, insulted, harassed, stereotyped, or discriminated against; and having unfair assumptions made about you** | | | | |  |  |
| --- | --- | --- | --- | --- | --- | --- |
| **How often are you treated unfairly because you are Aboriginal or Torres Strait Islander …** | **Never** | **Hardly ever** | **Some times** | **Often** | **Very often** | **This doesn’t apply to me** |
| At work or on the job? | ⃝ | ⃝ | ⃝ | ⃝ | ⃝ | ⃝ |
| At home, by neighbours or at somebody else’s house? | ⃝ | ⃝ | ⃝ | ⃝ | ⃝ | ⃝ |
| At school, university or other academic setting? | ⃝ | ⃝ | ⃝ | ⃝ | ⃝ | ⃝ |
| While doing sporting, recreational or leisure activities? | ⃝ | ⃝ | ⃝ | ⃝ | ⃝ | ⃝ |
| By the police, security personnel, lawyers or in a court of law? | ⃝ | ⃝ | ⃝ | ⃝ | ⃝ | ⃝ |
| By doctors, nurses or other staff at hospitals or doctors’ surgeries? | ⃝ | ⃝ | ⃝ | ⃝ | ⃝ | ⃝ |
| By staff of government agencies like Centrelink? | ⃝ | ⃝ | ⃝ | ⃝ | ⃝ | ⃝ |
| By staff at restaurants, bars, shops, banks, motels, real estate agents, in taxis or when getting any other services? | ⃝ | ⃝ | ⃝ | ⃝ | ⃝ | ⃝ |
| By people on the street, at shopping centres, sporting events, concerts, nightclubs? | ⃝ | ⃝ | ⃝ | ⃝ | ⃝ | ⃝ |
| By other Aboriginal or Torres Strait Islander people? | ⃝ | ⃝ | ⃝ | ⃝ | ⃝ | ⃝ |
| At work or on the job? | ⃝ | ⃝ | ⃝ | ⃝ | ⃝ | ⃝ |
| At home, by neighbours or at somebody else’s house? | ⃝ | ⃝ | ⃝ | ⃝ | ⃝ | ⃝ |
| At school, university or other academic setting? | ⃝ | ⃝ | ⃝ | ⃝ | ⃝ | ⃝ |

Adapted from the first question of the MIRE. This measure does not refer to a specific time period for exposure to discrimination. The MIRE prompt was slightly modified, as shown here.

Table S2. Second version of discrimination instruments tested in the Mayi Kuwayu Study

| **Everyday discrimination** | | | | | | |
| --- | --- | --- | --- | --- | --- | --- |
| **Source** | **How often do these things happen to you?** | **Not at all** | **A little bit** | **A fair bit** | **A lot** | **Is it because you are Aboriginal or Torres Strait Islander?** |
| * | You are treated with less respect than other people. | ⃝ | ⃝ | ⃝ | ⃝ | ⬜ Yes |
| *^†^ | You are given worse service than other people (for example at restaurants, stores, Centrelink, or from a mechanic) | ⃝ | ⃝ | ⃝ | ⃝ | ⬜ Yes |
| * | People act like they think you are not smart. | ⃝ | ⃝ | ⃝ | ⃝ | ⬜ Yes |
| * | People act like they are afraid of you. | ⃝ | ⃝ | ⃝ | ⃝ | ⬜ Yes |
| * | People act like you are not honest. | ⃝ | ⃝ | ⃝ | ⃝ | ⬜ Yes |
| * | People act like they are better than you. | ⃝ | ⃝ | ⃝ | ⃝ | ⬜ Yes |
| * | You are called names, insulted, or yelled at. | ⃝ | ⃝ | ⃝ | ⃝ | ⬜ Yes |
| * | You are threatened or harassed. | ⃝ | ⃝ | ⃝ | ⃝ | ⬜ Yes |
| ^‡^ | You are followed around in stores. | ⃝ | ⃝ | ⃝ | ⃝ | ⬜ Yes |
| ^§^ | You are watched more closely than others at work or school. | ⃝ | ⃝ | ⃝ | ⃝ | ⬜ Yes |
| ^†^ | Teachers tell you not to bother getting an education. | ⃝ | ⃝ | ⃝ | ⃝ | ⬜ Yes |
| ^†^ | Police unfairly bother you. | ⃝ | ⃝ | ⃝ | ⃝ | ⬜ Yes |
| ^†^ | You have trouble renting or buying an apartment or house. | ⃝ | ⃝ | ⃝ | ⃝ | ⬜ Yes |
|  |  |  |  |  |  |  |
|  | How stressful is it when these things happen? | ⃝ | ⃝ | ⃝ | ⃝ |  |
|  | How much have these things affected your life? | ⃝ | ⃝ | ⃝ | ⃝ |  |
| **Healthcare discrimination** | | | | | | |
| **Source** | **How often do these things happen to you when you receive health care?** | **Not at all** | **A little bit** | **A fair bit** | **A lot** | **Is it because you are Aboriginal or Torres Strait Islander?** |
| ^\|^ | Doctors and nurses do not listen to what you say. | ⃝ | ⃝ | ⃝ | ⃝ | ⬜ Yes |
| ^¶^ | People speak over you. | ⃝ | ⃝ | ⃝ | ⃝ | ⬜ Yes |
| ^\|^ | People act like they are better than you. | ⃝ | ⃝ | ⃝ | ⃝ | ⬜ Yes |
| ^¶^ | You have to wait longer than other people. | ⃝ | ⃝ | ⃝ | ⃝ | ⬜ Yes |
| ^¶^ | You do not receive the health care you need. | ⃝ | ⃝ | ⃝ | ⃝ | ⬜ Yes |
| ^\|^ | You receive poorer health care than other people. | ⃝ | ⃝ | ⃝ | ⃝ | ⬜ Yes |
| ^¶^ | You are sent home (discharged) without complete care or other arrangements (for example, transport home). | ⃝ | ⃝ | ⃝ | ⃝ | ⬜ Yes |
|  |  |  |  |  |  |  |
|  | How stressful is it when these things happen? | ⃝ | ⃝ | ⃝ | ⃝ |  |
|  | How much have these things affected your life? | ⃝ | ⃝ | ⃝ | ⃝ |  |

No time frame was specified within the measure for the experiences of discrimination. Formatting slightly modified from actual survey instrument. * Everyday Discrimination Scale. † Major Experiences of Discrimination. ‡ Expanded Everyday Discrimination Scale. § Chronic Work Discrimination and Harassment. | Discrimination in Medical Settings Scale. ¶ developed based on literature and/or personal experiences. The questions about perceived impact are adapted from “S16-17 of the Major Experiences of Discrimination: 9 item version from the MIDUS Study”, using the standard Mayi Kuwayu Study response options

Table S3. Distribution of the sample by demographic factors (N=6775), and distribution of the national Aboriginal and Torres Strait Islander adult population ≥15 years

|  | Mayi Kuwayu Study sample |  | National Aboriginal and Torres Strait Islander population ≥15 years |
| --- | --- | --- | --- |
|  | % (n) |  | % (n) |
| Indigenous identification |  |  |  |
| Aboriginal | 91.8 (6221) |  | 91.4 (479024) |
| Torres Strait Islander | 3.1 (210) |  | 5.1 (26549) |
| Both Aboriginal and Torres Strait Islander | 4.0 (271) |  | 3.5 (18459) |
| Missing | 1.1 (73) |  | -- |
| Age group* |  |  |  |
| 16-25 years | 10.3 (695) |  | 29.1 (160537) |
| 26-35 years | 11.9 (809) |  | 22.2 (122884) |
| 36-45 years | 14.0 (950) |  | 15.9 (87854) |
| 46-55 years | 20.4 (1381) |  | 15.2 (84163) |
| 56-65 years | 25.4 (1719) |  | 10.5 (57975) |
| ≥66 years | 18.0 (1221) |  | 7.1 (39019) |
| Gender |  |  |  |
| Male | 38.6 (2615) |  | 49.4 (272913) |
| Female | 61.3 (4155) |  | 50.6 (279519) |
| Other gender | 0.1 (5) |  |  |
| Level of remoteness* |  |  |  |
| Major Cities | 45.8 (3100) |  | 38.0 (209919) |
| Inner Regional areas | 28.8 (1953) |  | 42.9 (237001) |
| Outer regional areas | 18.1 (1223) |  |  |
| Remote and very remote areas | 7.4 (499) |  | 19.1 (105512) |

Data are based on the 2018 Aboriginal and Torres Strait Islander population projections for adults aged ≥15 years and over (extracted from ABS Stat, N=552432);^7^ data are not available for ≥16 years to align with the sample), with the exception of data on Indigenous identification, which is drawn from the 2016 ABS Census (N=524032).^8^

* Data from ABS Stat are provided for different age groups (15-24, 25-34, 35-44, 45-54, 55-64, ≥65 years) and remoteness categories (Major Cities, Inner Regional Areas and Outer Regional Areas combined) than the data from the Mayi Kuwayu Study.

Table S4. Mean total score for everyday and healthcare discrimination, by age group, gender, and remoteness (N=6775)

|  | **Mean total score (95CI)** | | | |
| --- | --- | --- | --- | --- |
|  |  |  |  |  |
|  | **Everyday discrimination** |  |  | **Healthcare discrimination** |
| **Age group (years)***^†^ |  |  |  |  |
| 16-25 | 3.40 (3.07,3.74) |  |  | 1.05 (0.91,1.18) |
| 26-35 | 3.23 (2.93,3.53) |  |  | 1.17 (1.02,1.32) |
| 36-45 | 3.61 (3.30,3.91) |  |  | 1.39 (1.23,1.56) |
| 46-55 | 3.43 (3.17,3.69) |  |  | 1.24 (1.12,1.36) |
| 56-65 | 2.29 (2.10,2.40) |  |  | 1.02 (0.92,1.12) |
| ≥66 | 1.19 (1.04,1.33) |  |  | 0.53 (0.45,0.61) |
|  |  |  |  |  |
| **Gender** |  |  |  |  |
| Male | 2.84 (2.66,3.01) |  |  | 1.01 (0.93,1.10) |
| Female | 2.70 (2.58,2.83) |  |  | 1.08 (1.01,1.14) |
| Other | -- |  |  | -- |
|  |  |  |  |  |
| **Level of remoteness***^†^ |  |  |  |  |
| Major city | 2.86 (2.70,3.01) |  |  | 1.03 (0.96,1.11) |
| Inner regional | 2.53 (2.35,2.72) |  |  | 0.97 (0.88,1.06) |
| Outer regional | 2.63 (2.39,2.87) |  |  | 1.05 (0.93,1.16) |
| Remote and very remote | 3.30 (2.89,3.71) |  |  | 1.53 (1.31,1.76) |

* Indicates significant differences in the mean everyday discrimination score across exposure levels (p-value for F statistic <0.05).
^†^ Indicates significant differences in the mean healthcare discrimination score across exposure levels (p-value for F statistic <0.05).
Due to small numbers of persons identifying as other gender, this category is included in totals but is omitted from gender-stratified and adjusted analysis.

**REFERENCES**

1. Field A. Discovering statistics using IBM SPSS statistics: And sex, drugs and rock 'n' roll. 4th ed. Singapore: Sage 2013.

2. Tabachnick BG, Fidell LS. Using Multivariate Statistics. 5th ed. Sydney: Allyn & Bacon/Pearson Education; 2007.

3. Boateng GO, Neilands TB, Frongillo EA, Melgar-Quiñonez HR, Young SL. Best practices for developing and validating scales for health, social, and behavioral research: a primer. *Front Public Health* 2018; **6**: 149.

4. Hu Lt, Bentler PM. Cutoff criteria for fit indexes in covariance structure analysis: Conventional criteria versus new alternatives. *Struct Equ Modeling* 1999; **6**(1): 1-55.

5. Bentler PM. Comparative fit indexes in structural models. *Psychol Bull* 1990; **107**(2): 238.

6. Maydeu-Olivares A, Shi D, Rosseel Y. Assessing Fit in Structural Equation Models: A Monte-Carlo Evaluation of RMSEA Versus SRMR Confidence Intervals and Tests of Close Fit. *Struct Equ Modeling* 2018; **25**(3): 389-402.

7. Australian Bureau of Statistics. Dataset: Projected population, Aboriginal and Torres Strait Islander Australians, Remotness Area, 2016 to 2031. Available from: <http://stat.data.abs.gov.au/index.aspx?DatasetCode=ABORIGINAL_POP_PROJ_REMOTE> [Accessed 16 November 2020]. Canberra, ACT: ABS; 2019.

8. Australian Bureau of Statistics. Estimates of Aboriginal and Torres Strait Islander Australians, June 2016 [Table 6]. Available from: <https://www.abs.gov.au/statistics/people/aboriginal-and-torres-strait-islander-peoples/estimates-aboriginal-and-torres-strait-islander-australians/latest-release#data-download> [Accessed 16 November 2020]. Canberra, ACT: ABS; 2018.
